# Serum Cholinesterases, a Novel Marker of Clinical Activity in Inflammatory Bowel Disease: A Retrospective Case-Control Study

**DOI:** 10.1155/2020/4694090

**Published:** 2020-07-14

**Authors:** Xiaona Shao, Lei Yang, Keyue Hu, Ruiwei Shen, Qunqun Ye, Xiaogang Yuan, Qiang Zhao, Jianwei Shen

**Affiliations:** ^1^Department of Gastroenterology, Ningbo Medical Center Lihuili Hospital, Ningbo, 315040 Zhejiang Province, China; ^2^Department of Intensive Care Unit, Ningbo Medical Center Lihuili Hospital, Ningbo, 315040 Zhejiang Province, China; ^3^Department of Hematology and Oncology, Hwa Mei Hospital, University of Chinese Academy of Sciences, Ningbo, 315010 Zhejiang Province, China

## Abstract

**Background:**

The aim of our study was to investigate whether serum cholinesterase (ChE) levels were associated with inflammatory bowel disease (IBD).

**Materials and Methods:**

We conducted a retrospective case-control study to clarify the relationship between serum ChE levels and IBD that included 142 patients with ulcerative colitis (UC), 60 patients with Crohn's disease (CD), and 264 healthy controls (HCs). We used ROC curves to evaluate the diagnostic value of serum ChE levels for IBD.

**Results:**

Substantially lower serum ChE levels were detected in patients with UC than in HCs (6376 U/L versus 8418 U/L, *P* < 0.001) and in patients with CD than in HCs (5181 U/L versus 8418 U/L, *P* < 0.001). Additionally, patients with CD displayed significantly lower serum ChE levels than patients with UC (5181 U/L versus 6376 U/L, *P* < 0.01). We also found that there was a negative association between serum ChE levels and the Crohn's Disease Activity Index (CDAI) score of patients with CD (*P* = 0.011) and the Simple Clinical Colitis Activity Index (SCCAI) score of patients with UC (*P* = 0.018). The area under the curve (AUC) for serum ChE for the diagnosis of IBD was 0.826, and the AUCs of serum ChE for the diagnosis of CD and UC were 0.890 and 0.800, respectively.

**Conclusions:**

Serum ChE levels have important clinical significance in the diagnosis and assessment of clinical activity in patients with IBD, and the cholinergic anti-inflammatory pathway may provide new ideas for targeted treatment of IBD.

## 1. Introduction

Inflammatory bowel disease (IBD), which includes Crohn's disease (CD) and ulcerative colitis (UC), is an idiopathic disease involving chronic inflammation of the gastrointestinal tract [1]. Although the pathogenesis of IBD has not yet been elucidated, most researchers currently believe that it is due to the interaction of genetic susceptibility, environmental factors, microbial flora imbalance, and immune disorders [2].

Cholinesterase (ChE) is a type of glycoprotein synthesized by the liver that can be divided into acetylcholinesterase (AChE) and butyrylcholinesterase (BChE). In recent years, BChE and AChE levels have been considered to be related to the occurrence and development of IBD and can be used as prognostic factors in some analyses [3–5]. Although there is an increasing incidence of IBD in China, the correlation between serum ChE levels and the risk of IBD has not been well elucidated in Chinese patients. Hence, the objective of our study was to investigate whether serum ChE levels were associated with IBD activity.

## 2. Materials and Methods

### 2.1. Ethical Considerations

The study protocol was approved by the ethics committee of Ningbo Medical Center Lihuili Hospital, Zhejiang, China.

### 2.2. Subjects

In this retrospective case-control study, we included a total of 60 patients with CD and 142 patients with UC who were admitted to the Department of Gastroenterology of the Ningbo Medical Center Lihuili Hospital from January 2007 to December 2018. UC and CD were diagnosed on the basis of standardized clinical, radiological, endoscopic, and pathological criteria, as described by Lennard-Jones [6]. The Crohn's Disease Activity Index (CDAI) score and the Simple Clinical Colitis Activity Index (SCCAI) score were used to assess clinical activity of patients with CD and UC [7, 8], respectively, which were categorized into the active phase (CDAI: total scores ≥5; SCCAI: total scores ≥3) and the remission phase (CDAI: total scores ≤4; SCCAI: total scores ≤2). In our study, we used a simpler version of the CDAI [7, 9], which was designed by Harvey and Bradshaw. A total of 264 healthy individuals who were free from chronic diseases and came from the same geographic area as the IBD group were chosen as the control group. Patients with indeterminate colitis and those diagnosed with IBD in whom UC and CD unable to be distinguished were excluded. In addition, patients with hepatobiliary disease, malignancy, or any other chronic infection that may affect serum ChE levels were also excluded.

We retrieved laboratory tests from the electronic medical record system, including serum ChE, serum albumin (ALB), calcium ions (Ca^2+^), platelets (PLT), and C-reactive protein (CRP). In our study, serum BChE was measured as the serum ChE level. Moreover, the clinical information of IBD patients was also collected, including age, sex, body mass index, clinical manifestations, medications, radiological performance, colonoscopy, and histopathological results.

ChE was measured by a butyrylthiocholine kit for cholinesterase assay (Ningbo Saike Biotechnology Co., Ltd., Ningbo, China) and an ADVIA Workcell (Siemens, Erlangen, Germany) autoanalyser.

### 2.3. Statistical Analysis

Statistical analyses were performed using SPSS version 23.0 (IBM SPSS Statistics, Chicago, USA) and GraphPad Prism 8 (GraphPad Software, California, USA). Normally distributed variables were expressed as the means ± the standard deviations (SDs), and nonnormally distributed variables were expressed as medians (interquartile ranges). Noncontinuous variables were denoted by the numbers and percentages. The Kolmogorov-Smirnov test was used to check for the normality of continuous variables. A one-way analysis of variance (ANOVA) followed by an LSD post hoc test was utilized for normally distributed variables among three or more groups. The Mann-Whitney test was employed for comparisons of nonnormally distributed variables. The chi-squared test was utilized for categorical variables. Correlations were determined using Pearson's coefficient to determine the association between serum ChE and clinical parameters. Receiver operating characteristic (ROC) analysis was used to determine the area under the curve (AUC) for the optimal cut-off value of each variable. A *P* value < 0.05 was considered statistically significant.

## 3. Results

### 3.1. Clinical Characteristics of IBD Patients and Healthy Controls (HCs)

Our study included 60 patients with CD, 142 patients with UC, and 264 HCs. The characteristics of patients with IBD and HCs are shown in Table 1. Analysis of serum ChE levels revealed no significance within phenotype characteristics of patients with CD or UC (Table 2).

### 3.2. Serum ChE in IBD Patients and HCs

**Figure 1:**
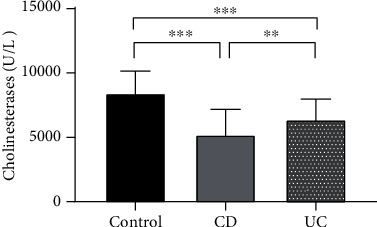
Serum ChE levels in CD patients, UC patients, and healthy controls. ^∗∗^*P* < 0.01; ^∗∗∗^*P* < 0.001.

Serum ChE levels were significantly lower in patients with CD (5181.87 ± 2094.99 U/L) and in those with UC (6376.51 ± 1713.76 U/L) than in HCs (8418.28 ± 1840.88 U/L) (*P* < 0.001). At the same time, the serum ChE levels of patients with CD and those with UC were also significantly different (*P* < 0.01) (see Figure 1).

### 3.3. Association of Clinical Indicators with Serum ChE

**Figure 2:**
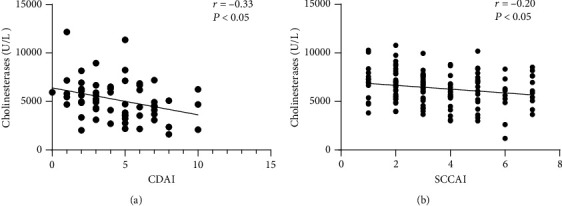
(a) The correlation between serum ChE levels and CDAI score in CD patients by using a scatter plot. (b) The correlation between serum ChE levels and SCCAI score in UC patients by using a scatter plot.

**Figure 3:**
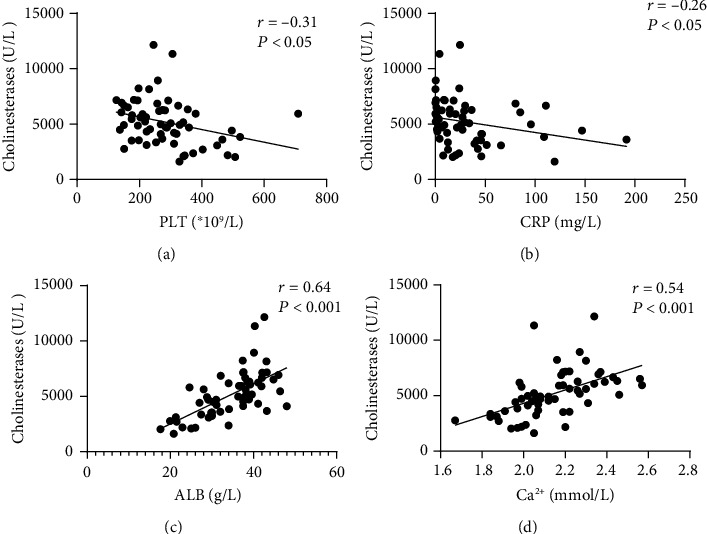
The correlation between serum ChE levels and (a) PLT, (b) CRP, (c) ALB, and (d) Ca^2+^ level in CD patients by using a scatter plot.

**Figure 4:**
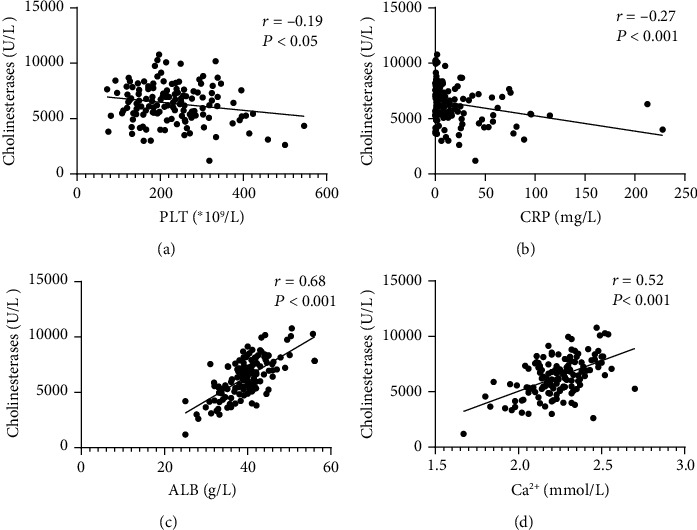
The correlation between serum ChE levels and (a) PLT, (b) CRP, (c) ALB, and (d) Ca^2+^ level in UC patients by using a scatter plot.

Serum ChE levels were negatively associated with the CDAI in patients with CD (*r* = −0.33, *P* < 0.05). Similarly, there was a negative association between serum ChE levels and the SCCAI in patients with UC (*r* = −0.20, *P* < 0.05, see Figure 2). Moreover, serum ChE levels were found to be negatively correlated with PLT and CRP levels and positively correlated with ALB and Ca^2+^ levels in patients with IBD (see Figures 3 and 4).

### 3.4. Value of Serum ChE in the Diagnosis of IBD (See Figure 5)

The area under the curve (AUC) of serum ChE for the diagnosis of IBD was 0.826; the optimal cut-off value of serum ChE was ≥7356 U/L, the sensitivity was 78.7%, the specificity was 72.7%, and the Jordan index was 0.514. The AUC of serum ChE for the diagnosis of CD was 0.890; the optimal cut-off value of serum ChE was ≥7204 U/L, the sensitivity was 91.7%, the specificity was 75.0%, and the Jordan index was 0.667. The AUC of serum ChE in the diagnosis of UC was 0.800; the optimal cut-off value of serum ChE was ≥7356 U/L, the sensitivity was 73.2%, the specificity was 72.7%, and the Jordan index was 0.460.

## 4. Discussion

Our study found that serum ChE levels in IBD patients were significantly lower than those in the control group, and with the increase in the IBD activity index (CDAI, SCCAI), the serum ChE levels decreased more significantly. Moreover, we proposed the first optimal cut-off values of serum ChE levels for the diagnosis of CD and UC, which both provided good evaluation values for the clinical diagnosis and judgement of the severity of IBD.

IBD, as a chronic nonspecific inflammatory reactive disease, involves a variety of immune cells and inflammatory factors. As a classic neural circuit that can regulate innate immunity, the cholinergic anti-inflammatory pathway is involved in the occurrence and development of IBD [10, 11]. Inflammation triggers an afferent vagal response that is transmitted to the hypothalamus, where it stimulates the efferent vagal nerve to release the neurotransmitter acetylcholine (ACh) [12]. ACh from the vagus tissue activates its *α*7 nicotinic acetylcholine receptor (*α*7nAChR) on macrophages, through which it intercepts the nuclear translocation of NF-*κ*B and inhibits the production of proinflammatory cytokines (TNF-*α*, IL-1*β*, IL-18, and IL-6). In addition, the use of nicotine in combination with *α*7nAChR can also ameliorate acute inflammation in IBD patients [13], which supports the importance of cholinergic stimulation as a modulator of IBD inflammation.

The regulation of systemic ACh levels requires continuous surveillance of the equilibrium between ACh production by the vagal nerve and its hydrolysis by AChE and BChE [12]. Because serum ChE levels reflect changes in the total ability of the body to hydrolyse Ach [12, 14], the decrease in serum ChE levels may be related to the increase in cholinergic anti-inflammatory pathways, resulting in a compensatory response resulting from the body's active downregulation of ChE activity.

In recent years, several studies have found that serum ChE levels are involved in a variety of diseases. Studies by Ben Assayag et al. [15] suggested that the decrease in serum ChE levels was a manifestation of the activation of cholinergic anti-inflammatory pathways in patients with cerebral infarction and that low serum ChE levels may indicate cholinergic crisis and low survival rates. Chen et al. [16] found that the level and activity of BChE in patients with poststroke dementia (PSD) were lower than those in poststroke patients without dementia, and they concluded that BChE may be helpful in the diagnosis of PSD. Seo et al. [17] analysed the prognostic significance of serum ChE in patients with acute decompensated heart failure (ADHF). They found that, compared with other objective nutritional indicators, ChE showed the best AUC value (0.745) to predict all-cause mortality. Hence, they believed that ChE was a powerful prognostic indicator that can predict all-cause mortality in patients with ADHF.

To further clarify our findings, we investigated mechanisms that may affect ChE activity. Maharshak et al. [12] found that microRNA 132 (miR-132) levels in inflammatory tissues of intestinal biopsies were significantly higher than those in noninflammatory tissues. While the role of miR-132 is to repress the translation of the local ChE mRNA, its increase reflects the body's compensatory performance by indirectly increasing the ACh concentration and thus enhancing the cholinergic anti-inflammatory pathway. In addition, the decrease in serum ChE levels may be due to strong intravascular inflammation, which leads to enhanced vascular permeability. Liver function was also impaired, which reduced the ability of the liver to synthesize ChE [18], resulting in lower serum ChE levels. Moreover, intestinal bleeding, malnutrition, and the loss of albumin may also contribute to the decrease in serum ChE levels in patients with IBD.

We also found that the serum ChE levels of patients with CD and those with UC were significantly different, which had not been reported before. Based on our statistical results of the ROC curve, the diagnostic accuracy of serum ChE for CD was higher than that for UC (the AUCs of serum ChE for the diagnosis of CD and UC were 0.890 and 0.800, respectively.). Hence, we further explored possible reasons. ChE is a common serum marker that reflects the nutritional status of patients [19]. It has been reported that low serum ChE levels are associated with liver damage, inflammation, and malnutrition [20]. CD has higher nutritional requirements than UC, which was confirmed in our study. In our study, we used body mass index to represent the nutritional status of IBD patients, and we found that body mass index in CD patients was lower than that in UC patients. CD is frequently associated with malabsorption and secondary protein-energy malnutrition [21]. Nutritional interventions for the treatment of CD were consistently effective but were limited and were generally not effective for UC patients [22, 23]. After nutritional therapy, serum BChE significantly improved in CD patients. On the other hand, the increased BChE levels after nutritional support demonstrated the recovery of nutritional status and liver protein synthesis [21]. Thus, we inferred that serum BChE levels may be more closely related to CD than to UC. Our study also found that serum ChE levels were positively correlated with human albumin and Ca^2+^ levels in UC and CD patients and were negatively correlated with platelet and CRP levels. A study by Krela-Kazmierczak et al. [24] confirmed that patients with IBD had lower calcium levels than controls. In the intestine, calcium is primarily absorbed in the duodenum and proximal jejunum [25]. Therefore, gastrointestinal disorders may result in poor calcium absorption [26]. They also found a negative correlation between steroid dose and calcium levels, which was confirmed by Huybers et al. [27], who showed that in mice orally administered methylprednisolone, duodenal absorption of Ca^2+^ was significantly decreased. Changes in the expression and activity of key Ca^2+^ transport proteins in the gut, induced by associated inflammatory mediators, may also be one of the causes of Ca^2+^ absorption disorders [25]. It has been reported [19] that commonly measured serum markers that reflect a patient's nutritional status include albumin and ChE. Serum albumin is produced in the liver, while serum BChE was also determined to be produced by the liver [3], which was consistent with our findings that serum levels of BChE were related to albumin levels. And we also found that serum ChE levels within phenotype characteristics revealed no significance in patients with CD or UC, from which we might infer that serum ChE levels were more relative to clinical activity than endoscopic finding.

Our study has some limitations. First, it was a retrospective case-control study and was, therefore, inevitably subject to bias. Second, due to the limitation of the number of patients, the sex and age composition ratios of the CD/UC/control group were different, which increased the effects of confounding factors on the results. Third, because of the lack of patient follow-up data, we did not explore the dynamic changes in serum ChE levels in patients with IBD. Fourth, we must admit that endoscopic activity has a crucial role in the evaluation of the diseases course, since there is no access to complete data regarding endoscopic activity throughout the 12-year study period, we used clinical indices (such as CD and UC scores) instead of endoscopic indices to assess IBD activity. The relationship between ChE level and endoscopic activity needs to be further studied. Therefore, the limitations of our study should be considered when interpreting the data.

## 5. Conclusion

Our results confirmed that, compared with healthy controls, serum ChE levels were decreased in patients with IBD, and the decline in serum ChE levels was more pronounced as clinical activity in IBD increased. We also found that the serum ChE levels of patients with CD and those with UC were significantly different. It needs to be further clarified the relationship between serum ChE and endoscopic activity in the future. Overall, serum ChE levels can be used as a simple, economical method to help diagnose and distinguish the different stages of IBD. With the increasing biomedical research on the cholinergic anti-inflammatory pathway and ChE, serum ChE is expected to become a new immunotherapy target for IBD.

## Figures and Tables

**Figure 5:**
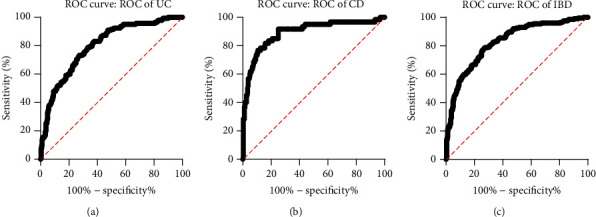
(a) ROC curve of serum ChE for UC patients. (b) ROC curve of serum ChE for CD patients. (c) ROC curve of serum ChE for IBD patients.

**Table 1:** Characteristics of patients with IBD and HCs.

Subjects	Patients with CD (*n* = 60)	Patients with UC (*n* = 142)	Healthy controls (*n* = 264)
Male/female (*n*)	39/21	56/86	170/94
Age (yr)	40.95 ± 16.88	52.02 ± 14.39	41.19 ± 16.65
ChE (U/L)	5181.87 ± 2094.99	6376.51 ± 1713.76	8418.28 ± 1840.88
CRP (mg/L)	31.93 ± 39.32	18.43 ± 33.57	/
ALB (g/L)	34.75 ± 7.36	39.67 ± 5.30	45.28 ± 4.43
PLT (^∗^10^9^/L)	283.00 ± 113.71	228.91 ± 87.24	201.83 ± 53.95
Ca^2+^ (mmol/L)	2.14 ± 0.19	2.24 ± 0.16	2.28 ± 0.14
Body mass index (kg/m^2^)	17.99 ± 1.95	22.51 ± 3.29	23.95 ± 3.27
Medications, *n* (%)			
5-ASA	42 (70%)	108 (76%)	/
Antibiotics	5 (8.3%)	22 (15.5%)	/
Steroids	13 (21.7%)	36 (25.3%)	/
Immunosuppression	8 (13%)	13 (9.2%)	/
Biological therapy	18 (30%)	4 (2.8%)	/

Data shown as mean ± standard deviation.

CD: Crohn's disease; UC: ulcerative colitis; ChE: cholinesterases; CRP: C-reactive protein; ALB: albumin; PLT: platelets.

**Table 2:** Relationship between phenotype characteristics and serum ChE levels of patients with IBD.

Subjects	*n* (%)	Serum ChE (*μ*mol/L)	*P*
CD (*n* = 60)			
Age at diagnosis (yr)			
A1 (≤16)	1 (1.7%)	5938	0.893
A2 (17-40)	31 (51.7%)	5251.45 ± 2415.56	
A3 (>40)	28 (46.7%)	5077.82 ± 1752.01	
Disease localization			
Ileal (L1)	22 (36.7%)	5021.86 ± 2125.72	0.821
Colonic (L2)	7 (11.7%)	5703.43 ± 1322.06	
Ileocolonic (L3)	27 (45.0%)	5267.93 ± 2367.43	
Isolated upper disease (L4)	4 (6.7%)	4568.25 ± 1106.70	
Disease behavior			
Nonstructuring/penetrating (B1)	40 (66.7%)	5441.28 ± 1884.60	0.076
Structuring (B2)	12 (20.0%)	5352.58 ± 2702.48	
Penetrating (B3)	8 (13.3%)	3628.75 ± 1592.71	
UC (*n* = 142)			
Disease extension			
Ulcerative proctitis (E1)	11 (7.7%)	7267.45 ± 1512.04	0.052
Left-sided UC (E2)	86 (60.6%)	6478.09 ± 1773.84	
Extensive (E3)	45 (31.7%)	5964.58 ± 1556.10	

Data shown as mean ± standard deviation. Phenotype assessed according to the Montreal classification.

## Data Availability

The data used to support the findings of this study were available from the corresponding author upon request.
